# The Impact of Complex Rehabilitation Treatment on Sarcopenia—Pathology with an Endocrine Morphological Substrate and Musculoskeletal Implications

**DOI:** 10.3390/medicina59071238

**Published:** 2023-07-02

**Authors:** Liliana-Elena Stanciu, Mădălina-Gabriela Iliescu, Carmen Oprea, Elena-Valentina Ionescu, Adina Petcu, Viorela Mihaela Ciortea, Lucian Cristian Petcu, Sterian Apostol, Andreea-Dalila Nedelcu, Irina Motoașcă, Laszlo Irsay

**Affiliations:** 1 Balneal and Rehabilitation Sanatorium of Techirghiol, 34-40 Dr. Victor Climescu Street, 906100 Techirghiol, Romania; lilianastanciu77@yahoo.com (L.-E.S.); carmen_oprea_cta@yahoo.com (C.O.); elena_valentina_ionescu@yahoo.com (E.-V.I.); sterianapostol@yahoo.com (S.A.); dalilanedelcu@yahoo.ro (A.-D.N.); 2 Department of Physical Medicine and Rehabilitation, Faculty of Medicine, “Ovidius” University of Constanta, 1 University Alley, Campus—Corp B, 900470 Constanta, Romania; 3 Faculty of Pharmacy, “Ovidius” University of Constanta, 1 University Alley, Campus—Corp B, 900470 Constanta, Romania; adinpetcu@yahoo.com; 4 Department of Rehabilitation Medicine, University of Medicine and Pharmacy “Iuliu Hatieganu”, 8 Victor Babes Street, 400012 Cluj-Napoca, Romania; motoascairina@gmail.com (I.M.); irsaylaszlo@gmail.com (L.I.); 5 Faculty of Dental Medicine, “Ovidius” University of Constanta, 7 Ilarie Voronca Street, 900178 Constanta, Romania; crilucpetcu@gmail.com

**Keywords:** sarcopenia, endocrine substrate, SARC-F, rehabilitation

## Abstract

The pathogenesis of sarcopenia is multifactorial, including changes in the endocrine system. Easy-to-perform screening tests can guide the diagnosis of sarcopenia and the rehabilitation therapeutic conduct, which can act on many physiopathological links. This study was conducted over a period of 5 months, from April to August 2022, and included 84 patients hospitalized for a period of 10 days in the Balneal and Rehabilitation Sanatorium Techirghiol for complex physiotherapy, which included balneotherapy. In dynamics, both at admission and discharge, specific screening tests for sarcopenia (SARC-F questionnaire, grip strength, testing muscle strength at the level of the quadriceps, sit-to-stand tests (the time required for five consecutive rises and the number of rises performed in 30 s)) and the Visual Analogue Scale (VAS) for pain were performed. The study was conducted according to the norms of deontology and medical ethics. *Results*: A significant proportion of patients had a positive result in at least one of the tests for the screening of sarcopenia syndrome. The most eloquent results were obtained from the statistical analysis of the following parameters evaluated at admission: the SARC-F questionnaire and the sit-to-stand test—the number of rises in 30 s. In terms of dynamics, after performing the complex rehabilitation treatment, the patients recorded improvements in the established screening tests and improvements in pain symptoms evaluated with the help of the VAS. *Conclusions*: Sarcopenia, a pathology developed with aging, is frequently encountered among adults. In the future, it is important to perform screening for sarcopenia in both endocrinology and medical rehabilitation clinics. Good management of sarcopenia can influence therapeutic conduct and can prevent complications, improving the functional capacity and the quality of life of the patients.

## 1. Introduction

The first hypothesis of the study is that sarcopenia is a frequent but underdiagnosed pathology among adults and the elderly, which is why performing scientifically validated screening tests is very important. The second study hypothesis supports the endocrine physiopathological substrate of sarcopenia and the possibility of obtaining a positive effect of the complex rehabilitation treatment in optimizing the functionality of the endocrine glands and the activity of proinflammatory cytokines.

Sarcopenia syndrome is characterized by the progressive loss of muscle mass, strength and function, starting with the 4th decade of life, in older adults [1]. It is a frequent pathology, and its prevalence increases with the aging of the population [2]. This syndrome represents a health problem that is frequently undiagnosed and has an impact on morbidity, mortality and healthcare expenditure [3]. The causes are multifactorial, involving the hormonal system, endocrinological decline, fatty infiltration, poor nutrition, alcohol consumption, smoking, the activity of the inflammatory pathway, neurological decline, loss of neuromuscular junctions and chronic diseases [1,4]. Sarcopenia is an underestimated and underdiagnosed pathology among geriatric patients, especially in the case of those with a history of stroke or malnutrition [5,6].

The consequences of sarcopenia are fatigue, falls, fractures, loss of function, frailty, loss of independence and disability [1]. For the good management of the progressive loss of muscle mass, strength and function, personalized and effective strategies for the prevention, diagnosis from an early stage and treatment of sarcopenia must be adopted [2]. To improve the functional status, the management of sarcopenia focuses on increasing physical activity and ensuring adequate nutrition, even from adulthood [1,7].

Since 2016, sarcopenia has been recognized as a disease entity under the following diagnostic code: ICD_10_M62.8 [8,9]. Measuring muscle mass is a cornerstone in establishing the diagnosis. This measurement can be performed by dual-energy X-ray absorptiometry (DXA), magnetic resonance imaging (MRI) and computed tomography (CT) [10]. Additionally, morphological estimates of skeletal muscle can be based on anthropometric measurements or, in a more modern approach, on ultrasound measurements [4,8].

These methods can be expensive and time-consuming and may require experienced medical personnel, which is why screening tests must be performed beforehand. Some useful screening tools will be described in the present study.

The pathogenesis of sarcopenia can be supported by the increased levels of tumor necrosis factor alpha (TNF-α) that occur with aging, being associated with the loss of muscle fibers and apoptosis [11]. Also, high concentrations of interleukin 6 (IL-6) may play an important role in modulating the inflammatory pathway during skeletal muscle loss [12].

The changes in the endocrine system that occur with age could be trigger factors for sarcopenia because the endocrine system has complex interactions with skeletal muscles, contributing to muscle development and the modulation of muscle strength [13]. The decline of hormones that maintain muscle (insulin-like growth factor (IGF-1), dehydroepiandrosterone sulfate (DHEA-S), testosterone, estrogen) can contribute to the appearance of sarcopenia [13]. The hypothalamic–pituitary–adrenal axis becomes dysfunctional with age, causing the adrenal cortex to release more glucocorticoids that induce a decrease in protein synthesis by affecting myostatin and IGF-1 [13]. Vitamin D deficiency can increase the risk of sarcopenia. Several hypotheses claim that supplementation with vitamin D can be effective in reaching and treating this syndrome.

A study published in 2011 demonstrates that sapropelic mud therapy influences the serum level of proinflammatory cytokines. The patients selected in the study with increased values of TNF-α, IL-1β and IL-6 recorded normal values at discharge, after ten days of treatment [10]. 

The findings show that the clinical benefits of balneotherapy in musculoskeletal disease patients may be mediated by an anti-inflammatory effect. A decrease in circulating IL-6 levels was noticed, as were beneficial improvements in pain decrease and patient functionality [14].

Currently, due to the lack of knowledge and due to the multifactorial genesis, the approach for a patient with sarcopenia is not based on basic etiopathogenesis, and the manifestations of sarcopenia are frequently masked by the multiple comorbidities that elderly patients may have [13,15].

The main objectives of this study were to demonstrate the importance of screening tests and to evaluate the impact of multimodal rehabilitation treatment on patients with sarcopenia, a pathology with an endocrine morphological substrate.

## 2. Methods

### 2.1. Study Design

This was a prospective, longitudinal, before–after study conducted over a period of 5 months, from April to August 2022, in a representative unit in the field of medical rehabilitation in Romania.

### 2.2. Subjects

The study included 84 patients, aged between 50 and 79 years, hospitalized for 10 days in Techirghiol Balneal and Rehabilitation Sanatorium for balneary–physical–kinetic treatment.

The inclusion criteria for the patients were as follows:

- Hospitalized in Balneal and Rehabilitation Sanatorium Techirghiol for musculoskeletal disorders;
- Aged between 50 and 79 years;
- Conscious, cooperative;
- The possibility of performing all the pre-established screening tests.

The exclusion criteria for the patients were as follows:

- Outpatient treatment for musculoskeletal disorders;
- Age outside the selected range;
- Uncooperative patients;
- Comorbidities associated with the impossibility of performing all the pre-established screening tests.

### 2.3. Intervention

The patients were evaluated, by screening tests, in dynamics, both at admission and discharge to observe the impact of complex rehabilitation treatment on muscle mass, strength and function in the context of sarcopenia.

The rehabilitation treatment performed by the patients was standard, according to the existing treatment protocols at the level of the medical unit and the national and international balneological approach. The interventional treatment included 10 daily sessions of hydrokinetotherapy and hydrothermotherapy using specific natural environmental factors, namely sapropelic mud and salt water from Techirghiol Lake, as well as artificial physical agents such as electrotherapy, massage and of course exercise therapy.

The baths in the salty water of Lake Techirghiol (at 35 °C) and mud baths (at 38 °C) were alternated daily, for a period of 20 min. The hydrokinetotherapy was performed on the recommendation of the rehabilitation doctor and under the guidance and supervision of the rehabilitation assistant. At the recommendation of the rehabilitation doctor, each patient performed, depending on the associated musculoskeletal pathology, three electrotherapy procedures daily. Massage and exercise therapy were performed on the doctor’s recommendation by the rehabilitation assistant, daily. For each patient included in the study, the same therapeutic objectives were respected to obtain a similar functional impact.

### 2.4. Measures

**Figure 1:**
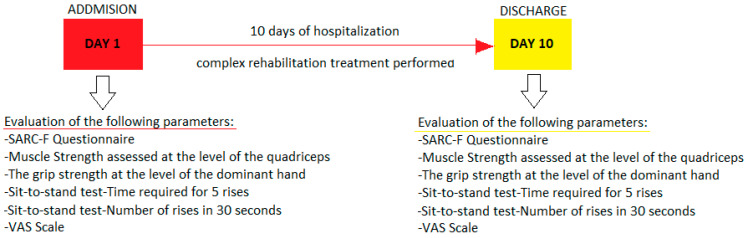
Protocol timeline of the study.

The SARC-F questionnaire, specific functional tests for sarcopenia and the Visual Analogue Scale were performed and analyzed. The study protocol is presented in Figure 1.

The SARC-F questionnaire is a screening tool, easy to use in daily practice, made up of 5 questions (strength, walking assistance, lifting from a chair, climbing stairs, falling) [3]. Both at admission and at discharge, on day 1 and day 10, the patients completed the questionnaire and evaluated each question with 0–2 points. A score greater than or equal to 4 gives a positive result in the screening test performed with the help of this tool [3].

Other sarcopenia screening tools that evaluate muscle strength and performance are represented by grip strength (assessed with a dynamometer), testing muscle strength at the level of the quadriceps, sit-to-stand tests (the time required for five consecutive rises and the number of rises performed in 30 s).

Both at admission and at discharge, a subjective evaluation method that depends on the evaluator was used to evaluate quadriceps strength. On the first day and last day of hospitalization, the patient performed a calf extension while sitting, with the knee flexed at 90° and the leg outside the table, while the doctor put resistance on the lower third of the calf. This test was performed to assess if the patient had low muscle strength at this level.

The grip strength was evaluated with the help of a digital hand dynamometer at the level of the dominant hand both at admission (day 1) and at discharge (day 10). Suggestive values for sarcopenia depend on the sex of the patient. Positive screening for sarcopenia was considered <16 kg for women and <27 kg for men [7].

Sit-to-stand tests were performed both at admission (first day) and at discharge (last day). The patients were asked to stand up and sit on a chair with feet about shoulder width apart and the arms crossed at the wrist and held against the chest. The time taken to rise from the chair 5 times was recorded. A value greater than 15 s provided a positive screening for sarcopenia [7]. The second test consisted in counting the rises performed in 30 s. The positive screening depended on the patient’s sex and age (Table 1).

In order to observe how many patients in the investigated group had a positive screening for sarcopenia, all five tests were performed.

The Visual Analogue Scale represents a subjective method for chronic and acute pain assessment [16]. This scale, which is easy and quick to perform, was applied to all the patients included in the study. To evaluate if the rehabilitation treatment improved the pain syndrome, patients were verbally asked by the clinician where the pain is on a scale from 0 (no pain) to 10 (worst pain imaginable), both at admission and at discharge.

### 2.5. Data Analysis

Each screening test performed at admission was analyzed as follows:

- In relation to certain fixed parameters (sex, environment, age, weight status);
- In relation to the Visual Analogue Scale (VAS) at admission/discharge;
- In relation to the other screening tests performed at admission;
- In relation to the result obtained in the same screening test at discharge (dynamic evaluation by performing a statistical association).

Statistical correlations were determined to identify the parameters of the screening tests that correlate best, as well as which screening test is more suggestive of sarcopenia.

The statistical analysis was performed using IBM SPSS statistics software version 23. The following tests were used in the statistical analysis of the obtained data: Wilcoxon signed-rank test and Z test for associations, chi-square test and Spearman’s rank-order for correlations.

The study protocol was designed in accordance with the ethical guidelines of the Declaration of Human Rights and was approved by the Techirghiol Balneal and Rehabilitation Sanatorium Ethics Committee.

## 3. Results

Of the 84 analyzed patients, 61% were women, 82.1% were from urban areas, and 47.6% were aged between 50 and 59 years. It was found that 85.71% of the investigated patients were positive in at least one screening test performed for sarcopenia. Statistically significant associations and correlations were observed between the performed screening tests and between them and the rest of the evaluated parameters.

### 3.1. Results Obtained According to Each Screening Test

**Figure 2:**
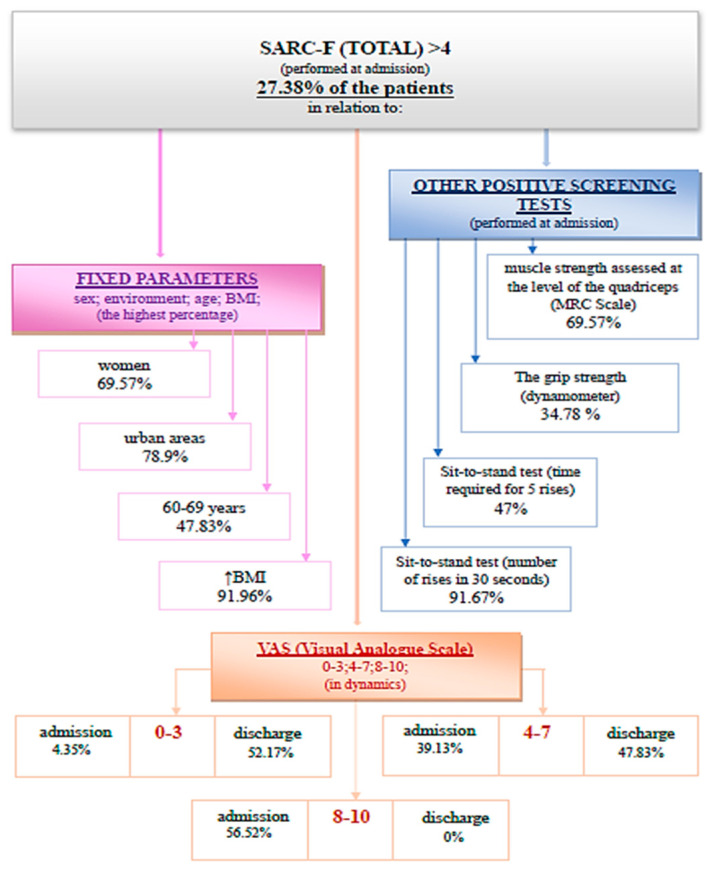
Schematic description of the SARC-F questionnaire (total), performed at admission.

(1) SARC-F questionnaire is suggestive of sarcopenia when a value higher than 4 is obtained. A percentage of 27.38% of patients had a positive screening for sarcopenia through the SARC-F questionnaire at admission (Figure 2).

**Figure 3:**
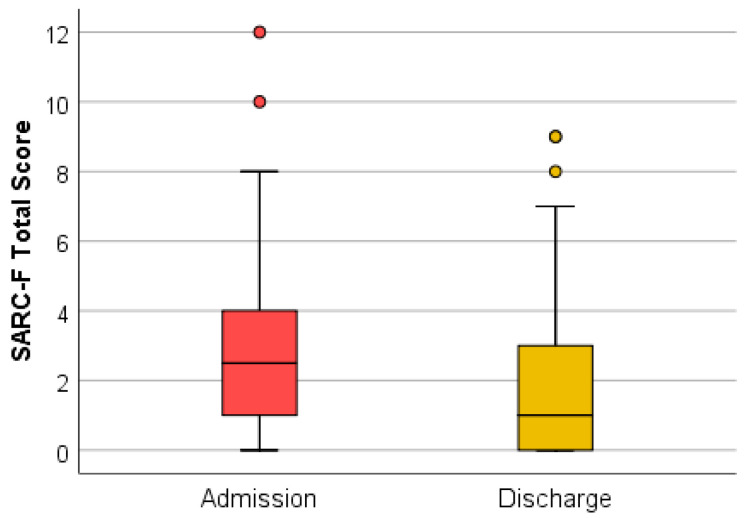
SARC-F (total) at admission and discharge.

The evaluation of the SARC-F questionnaire at discharge, after the complex rehabilitation treatment was performed, identified 13.10% of the total analyzed patients with a positive screening for sarcopenia. The association between SARC-F (total) at admission and discharge using the Wilcoxon signed-rank test and the Z test was statistically relevant (Figure 3).

The Wilcoxon signed-rank test determined that there was a statistically significant median decrease in SARC-F (total) score at admission (2.50) compared to SARC-F (total) score at discharge (1.00), Z = −5.456, *p* < 0.0001.

**Figure 4:**
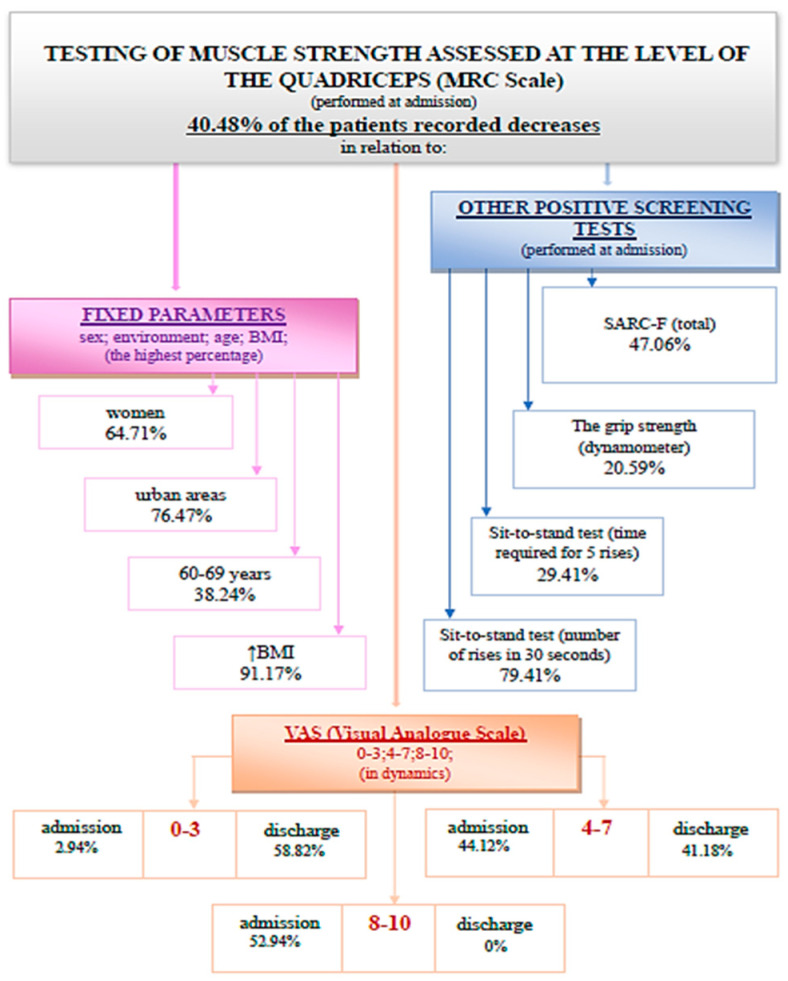
Schematic description of the muscle strength assessed at the level of the quadriceps (MRC Scale), performed at admission.

(2) The testing of muscle strength at the level of the quadriceps was performed according to the MRC scale (Medical Research Council Scale for Power of Muscle). A percentage of 40.48% of the evaluated patients recorded decreases in muscle strength (Figure 4).

**Figure 5:**
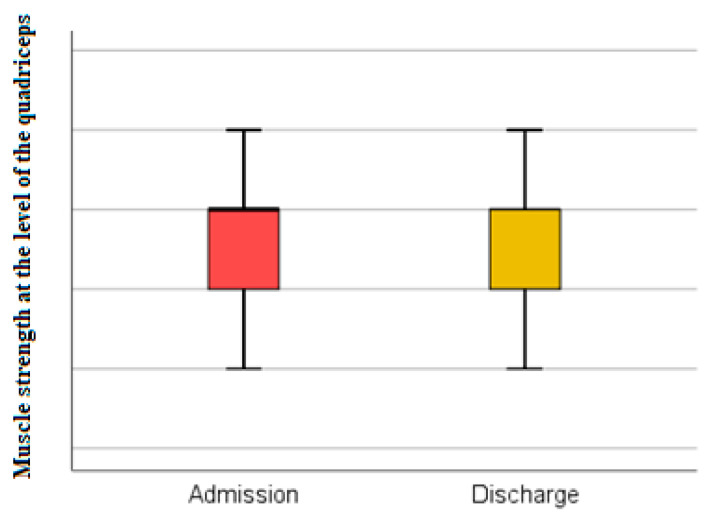
Muscle strength at the level of the quadriceps at admission and discharge.

The evaluation of muscle strength at the quadriceps level at discharge confirms that 30.95% of the total 84 patients registered a decrease in muscle strength. The association between quadriceps muscle strength at admission and discharge using the Wilcoxon signed-rank test and Z test has no statistical significance (Figure 5).

The Wilcoxon signed-rank test showed that at discharge, the treatment did not elicit a statistically significant change in testing muscle strength at the level of the quadriceps (Z = −1.633, *p* = 0.102). Indeed, the median score rating was the same at admission and at discharge.

**Figure 6:**
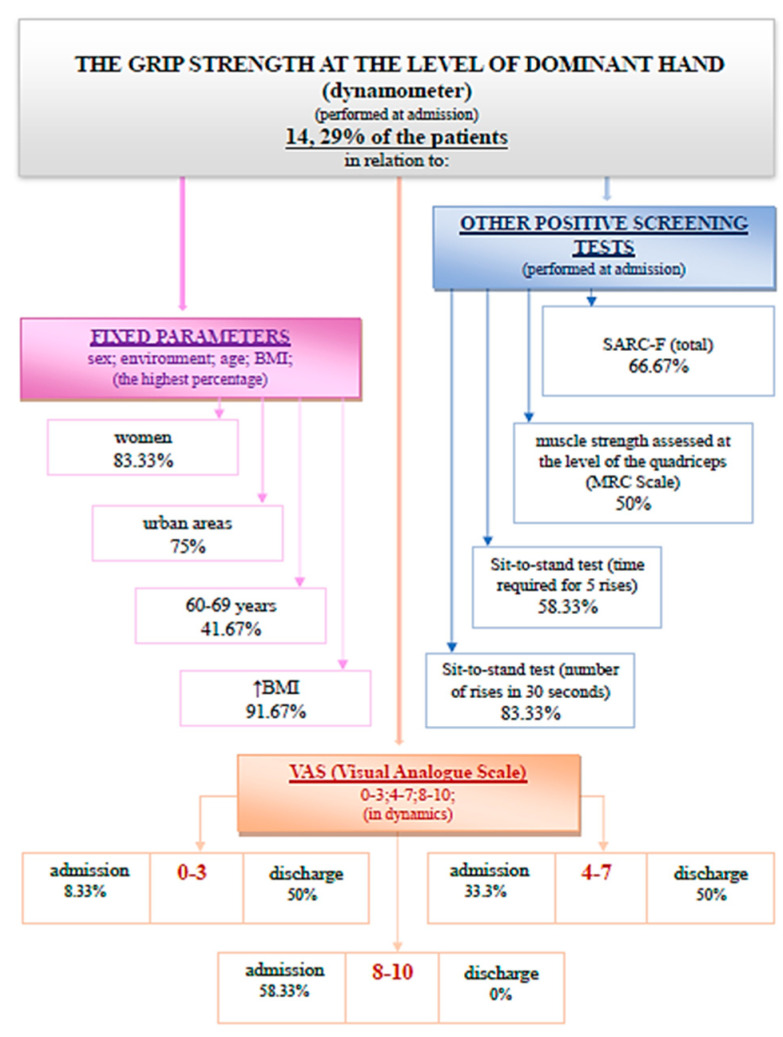
Schematic description of the grip strength at the level of dominant hand, performed at admission.

(3) The evaluation of the grip strength at the level of the dominant hand was performed with the help of a dynamometer. It is observed that 14.29% of the patients registered values compatible with sarcopenia at admission (Figure 6).

**Figure 7:**
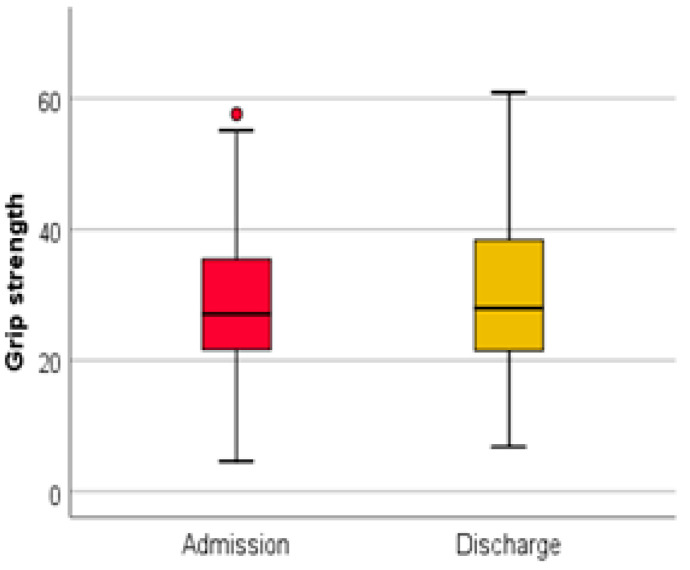
Grip strength at admission and discharge.

At discharge, 11.9% of patients had a positive screening for sarcopenia through this evaluation method. The association of the grip strength at admission and at discharge using the Wilcoxon signed-rank test and the Z test was statistically significant (Figure 7).

The Wilcoxon signed-rank test determined that there was a statistically significant median increase in grip strength (kg) at admission compared to grip strength (kg) at discharge, Z = −2.958, *p* < 0.0001.

**Figure 8:**
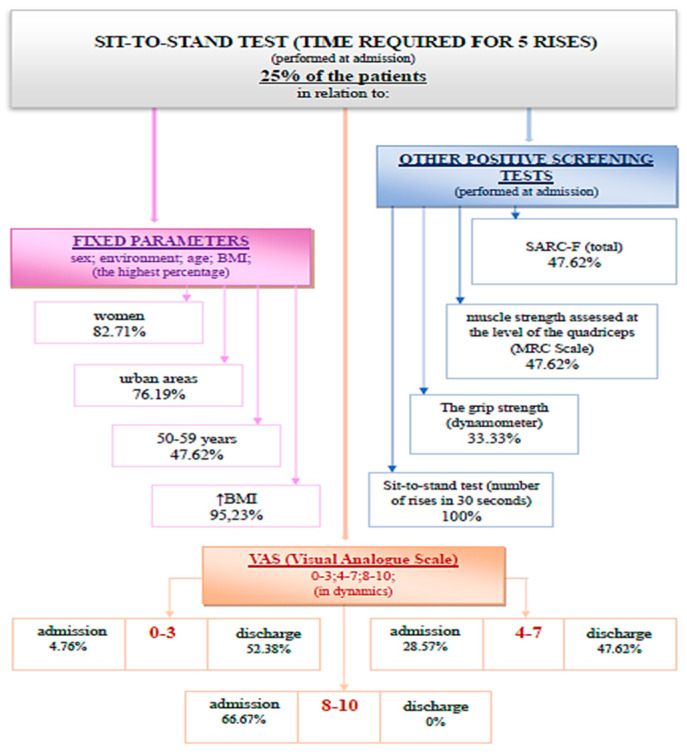
Schematic description of the sit-to-stand test (time required for 5 rises), performed at admission.

(4) Performing the sit-to-stand test (the time required for five consecutive rises) recorded a positive screening for sarcopenia (>15 s) in 25% of patients (Figure 8).

**Figure 9:**
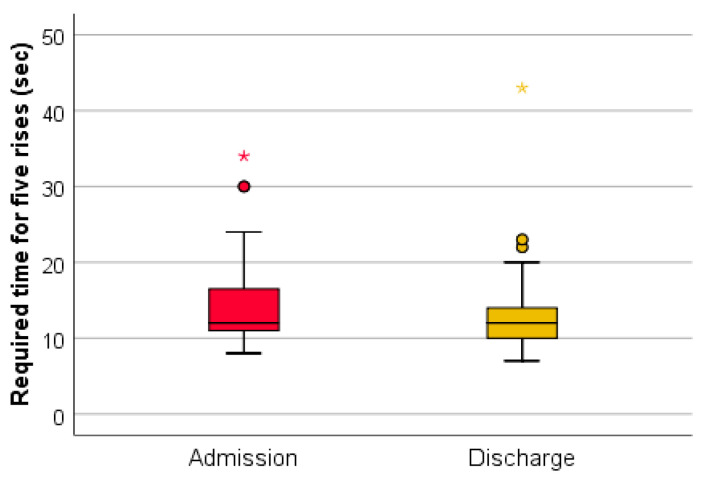
The time required for five consecutive rises at admission and discharge. */★—cohort.

Performing the sit-to-stand test (the time required for five consecutive rises) at discharge recorded values for sarcopenia in 14% of cases. The association of this parameter at admission and at discharge using the Wilcoxon signed-rank test and Z test had statistical significance (Figure 9).

The Wilcoxon signed-rank test determined that there was a statistically significant median decrease in the time required for five consecutive rises at admission (12.65) compared to the time required for five consecutive rises at discharge (12.00), Z = −4.468, *p* < 0.0001.

(5) The interpretation of the results of the sit-to-stand test (the number of rises in 30 s) depends on the patient’s sex and age (Table 1).

**Figure 10:**
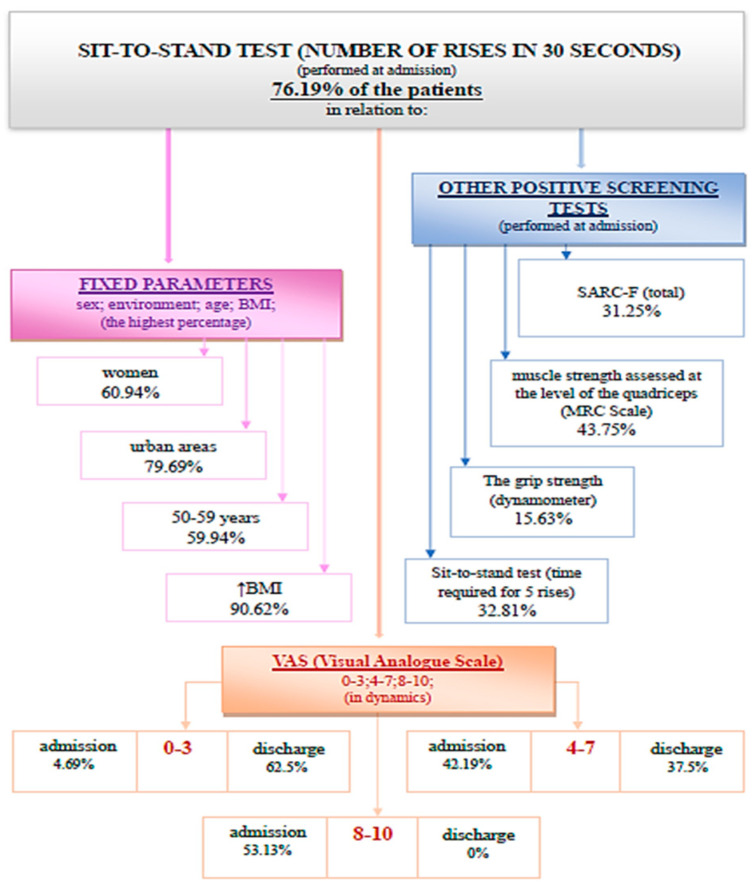
Schematic description of the sit-to-stand test (number of rises in 30 s), performed at admission.

This test used as a screening method for sarcopenia was positive for 76.19% of the 84 evaluated patients (Figure 10).

**Figure 11:**
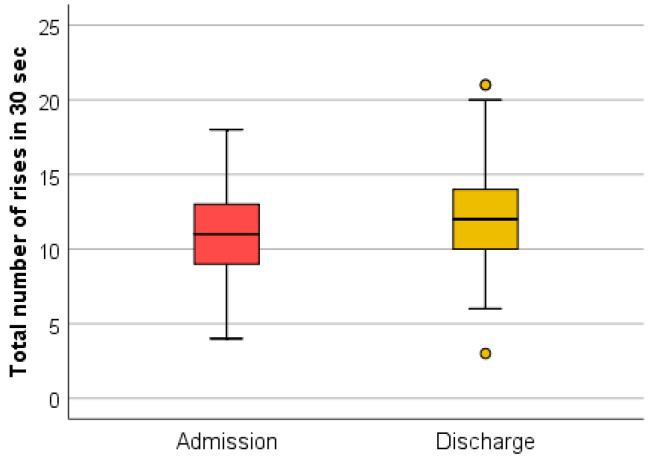
The sit-to-stand test (number of rises in 30 s) at admission and discharge.

At discharge, 57.14% of patients had a positive screening for sarcopenia through the sit-to-stand test (the number of rises in 30 s). The association of this parameter at admission and at discharge using the Wilcoxon signed-rank test and Z test had statistical significance (Figure 11).

The Wilcoxon signed-rank test determined that there was a statistically significant median increase in the sit-to-stand test (the number of rises in 30 s) at admission (11.00) compared to the sit-to-stand test (the number of rises in 30 s) at discharge (12.00), Z = −5.405, *p* < 0.0001.

### 3.2. VAS Scores in Dynamics (Statistical Association)

**Figure 12:**
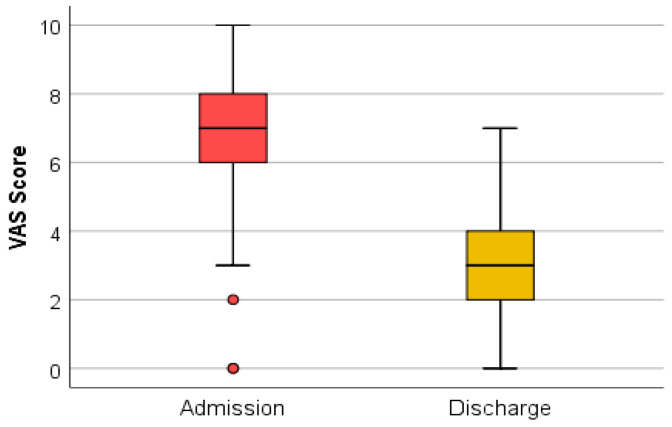
VAS score at admission and discharge.

The VAS scores obtained at admission and discharge were associated and evaluated using the Wilcoxon signed-rank test and the Z test. The results obtained showed that the algo-dysfunctional syndrome evaluated with the help of the Visual Analogue Scale (VAS), present at admission, improved during hospitalization, with significant relief of the pain (Figure 12).

The Wilcoxon signed-rank test determined that there was a statistically significant median decrease in VAS score at admission (7.00) compared to VAS score at discharge (3.00), Z = −7.960, *p* < 0.0001.

### 3.3. Relevant Statistical Correlations Identified between the Associated Parameters

(1) By performing chi-square tests, it was observed that there was a statistically significant association between sex and grip strength (kg) at admission (*p* = 0.000 < 0.05), sex and the sit-to-stand test (the time required for five consecutive rises (seconds)) at admission (*p* = 0.011 < 0.05) and sex and the sit-to-stand test (the number of rises in 30 s) at admission (*p* = 0.016 < 0.05) (Table 2, Table 3 and Table 4).

(2) By performing chi-square tests, it was observed that there was a statistically significant association between age and quadriceps muscle strength at discharge (*p* = 0.024 < 0.05) and between age and the sit-to-stand test (the number of rises in 30 s) at discharge (*p* = 0.002 < 0.05) (Table 5 and Table 6).

(3) By performing chi-square tests, relevant statistical correlations were identified between weight status and the sit-to-stand test (the number of rises in 30 s) at admission (*p* = 0.000 < 0.05).

(4) By performing chi-square tests and Spearman’s rank-order correlation, relevant statistical correlations were identified between the SARC-F (total) and the other established tests of screening.

Statistically significant associations were found between SARC-F (total) at admission and quadriceps muscle strength at admission (*p* = 0.002 < 0.05), SARC-F (total) at admission and grip strength (kg) at admission (*p* = 0.000 < 0.05), SARC-F (total) at admission and the sit-to-stand test (the time required for five consecutive rises (seconds)) at admission (*p* = 0.000 < 0.05) and SARC-F (total) at admission and the sit-to-stand test (the number of rises in 30 s) at admission (*p* = 0.013 < 0.05) (Table 7, Table 8, Table 9 and Table 10).

A Spearman’s rank-order correlation was run to determine the relationship between SARC-F (total) at admission and grip strength (kg) at admission/the sit-to-stand test (the time required for five consecutive rises (seconds)) at admission /the sit-to-stand test (the number of rises in 30 s) at admission.

There was a moderate, negative correlation between SARC-F (total) at admission and grip strength (kg) at admission, which was statistically significant (*p* < 0.001); a moderate, positive correlation between SARC-F (total) at admission and the sit-to-stand test (the time required for five consecutive rises (seconds)) at admission, which also was statistically significant (*p* < 0.001); and a moderate, negative correlation between SARC-F (total) at admission and the sit-to-stand test (the number of rises in 30 s) at admission, which also was statistically significant (*p* < 0.001) (Table 11).

There was a statistically significant association between SARC-F (total) at discharge and quadriceps muscle strength at discharge (*p* = 0.002 < 0.05), SARC-F (total) at discharge and grip strength (kg) at discharge (*p* = 0.001 < 0.05), SARC-F (total) at discharge and the sit-to-stand test (the time required for five consecutive rises (seconds)) at discharge (*p* = 0.000 < 0.05) and between SARC-F (total) at discharge and the sit-to-stand test (the number of rises in 30 s) at discharge (*p* = 0.001 < 0.05) (Table 12, Table 13, Table 14 and Table 15).

A Spearman’s rank-order correlation was run to determine the relationship between SARC-F (total) at discharge and grip strength (kg) at discharge/the sit-to-stand test (the time required for five consecutive rises (seconds)) at discharge/the sit-to-stand test (the number of rises in 30 s) at discharge.

There was a moderate, negative correlation between SARC-F (total) at discharge and grip strength (kg) at discharge, which was statistically significant (*p* < 0.001); a weak, positive correlation between SARC-F (total) at discharge and the sit-to-stand test (the time required for five consecutive rises (seconds)) at discharge, which also was statistically significant (*p* < 0.001); and a moderate, negative correlation between SARC-F (total) at discharge and the sit-to-stand test (the number of rises in 30 s) at discharge, which also was statistically significant (*p* < 0.001) (Table 16).

## 4. Discussion

It is important to have a screening strategy that allows the detection of sarcopenia in the early stages because it is extremely difficult to regain the already lost skeletal muscle mass. An ideal screening test must be useful, valid, reliable and also cost-effective. Screening strategies can be carried out through several tools, and it is necessary to identify which is the most effective way to detect sarcopenia and to choose some methods optimal for the prevention and treatment of this pathology [3].

The evaluation and diagnosis of sarcopenia can represent a new objective in defining the incidence and prevalence of this pathology, which is why it is necessary to establish a solid and comprehensive algorithm. Refinement of the algorithm and its use in current practice can represent a tool for monitoring progress [4].

The results of the study demonstrated that our objectives were achieved, the screening tests proved their usefulness in identifying sarcopenia, and rehabilitation treatment can have an effect on sarcopenia through the possible effects on the endocrine system.

To our knowledge, this is the only study using balneotherapy for sarcopenic patients. Other studies which applied physiotherapy as a therapeutic intervention had good results for exercise therapy, while other methods were not largely studied [3,15,17,18,19,20].

In this study, it was observed that although 85.71% of the patients were positive in at least one screening test, none were previously investigated for sarcopenia, and also none of them had a diagnosis of sarcopenia in their pathological history. A percentage of 27.38% of the evaluated patients had a positive screening for sarcopenia through the SARC-F questionnaire at admission. A percentage of 40.48% of the patients recorded decreases in the testing of muscle strength at the level of the quadriceps at admission. It is observed that 14.29% of the patients registered values compatible with sarcopenia in the evaluation of grip strength at the level of the dominant hand. The time required for five consecutive rises in the sit-to-stand test recorded a positive screening for sarcopenia for 25% of the patients. The number of rises in 30 s in the sit-to-stand test as a screening method for sarcopenia was positive for 76.19% of the evaluated patients.

The dynamic assessment of screening tests, both at admission and discharge, suggests that complex rehabilitation treatment can have an impact on sarcopenia. The decrease in the SARC-F (total) score at discharge indicates an improvement in functionality. This may be due to the decrease in the algo-functional syndrome and the increase in general muscle strength after performing the complex rehabilitation treatment. The testing of quadriceps muscle strength did not register considerable changes because this method is subjective and the improvements are difficult to quantify. The muscle strength measured with the dynamometer recorded a statistically significant increase. This method of evaluation uses a standard technique, devoid of subjectivity. The sit-to-stand tests (the time required for five consecutive rises and the number of rises in 30 s) were shortened at discharge, which indicates an increase in muscle strength and resistance following the complex treatment performed during hospitalization. These improvements can be due to both the hormonal implications (that complex rehabilitation treatment has through the use of natural therapeutic factors) and kinesiotherapy.

It was observed that evaluation of the Visual Analogue Scale (VAS) at discharge recorded improvements, with significant relief of the pain. Protocols that combine specific rehabilitation treatments with balneotherapy, including hydrotherapy, mud baths and exercise in hot thermal mud, could reduce pain and disability in patients identified as responders [21].

Relevant statistical correlations were identified between the associated parameters. The general muscle strength evaluated both at the level of the upper limbs and the lower limbs is related to sex. The endocrine system plays an important role, making the connection between the level of testosterone and estrogen in both sexes and the decline of muscle strength. There is a close relationship between age and muscle strength in the lower limbs. At discharge, the patients in the lower age ranges recorded improvements in the functional tests. This fact can be explained by hormonal status in relation to the patient’s age. Most of the investigated patients have an increased body mass index. The obtained results support the concept of sarcopenic obesity from the literature [22,23,24,25,26]. Thus, the excess representation of adipose tissue can influence functionality.

It was found that the SARC-F questionnaire correlates, both at admission and discharge, with all other screening tests. Considering this, in daily practice, only the SARC-F questionnaire and the number of rises performed in 30 s in the sit-to-stand test can be used for the screening of sarcopenia.

The tests indicated for the diagnosis of sarcopenia can be performed for all cooperative, conscious patients who have multiple associated diseases. Likewise, rehabilitation treatments that use artificial and natural physical agents can be recommended to patients with kidney, liver or heart damage, without determining the side effects of drug treatments [7,8,9,10,13,20,21] The physical factors recommended in physical and rehabilitation medicine are considered environmentally friendly alternatives compared to drugs for the treatment of various pathologies [7,10].

### 4.1. Limitations of the Study

Because the screening does not always confirm the diagnosis and more advanced diagnostic methods are needed, no control group was established. The lack of a control group can be considered a limitation of the study, along with the high costs that advanced sarcopenia diagnostic methods can have. Another limitation of the study is that no studies were found in the literature about sarcopenia and complex rehabilitation treatment that includes balneotherapy, which could have an impact on the endocrine system.

### 4.2. Applications and Suggestions for Future Research

In the future, performing screening tests in endocrinology and medical rehabilitation clinics can guide the establishment of diagnosis and treatment of sarcopenia. Even though there have been efforts to standardize a blood test kit for sarcopenia, there is currently no such kit in use in clinical settings [18]. For future studies, a larger number of patients, a control group and more diagnostic methods will be used.

## 5. Conclusions

The interpretation of our findings demonstrates that the results of the study support the hypothesis, that the screening tests, which are easy to perform routinely, prove their usefulness in the diagnosis of sarcopenia and that multimodal rehabilitation treatment has an effect on sarcopenia, improving the results of the screening tests.

The link that sarcopenia creates between endocrinology and medical rehabilitation is based on the hormonal changes that balneal treatment with natural environmental factors and kinesiotherapy can induce. The screening tests—SARC-F questionnaire, grip strength (assessed with a dynamometer), testing muscle strength at the level of the quadriceps, and sit-to-stand tests (the time required for five consecutive rises and the number of rises performed in 30 s)—are easy to perform in a short time, are reliable and have minimal costs. The main parameters analyzed at admission with statistically significant results were the SARC-F questionnaire and the number of rises performed in 30 s in the sit-to-stand test, which confirms the importance of screening by these methods in the evaluation of sarcopenia. Positive statistical variations following the therapeutic intervention were obtained for all the parameters included in the research, except the muscle strength at the level of the quadriceps, which underlines the importance of rehabilitation treatment in sarcopenia.

Screening can be easily performed in both endocrinology and medical rehabilitation clinics, and it can guide the conduct of diagnosis and treatment of sarcopenia (food supplements/hormonal supplements/increasing muscle strength with kinesiotherapy/balneal treatment with natural environmental factors). Sarcopenia has an endocrine morphological substrate with multiple functional implications. The approach to this pathology must be multidisciplinary.

## Figures and Tables

**Table 1:** Positive screening for sarcopenia by using sit-to-stand test—the number of rises in 30 s.

Age	Male	Female
50–54 years old	<16 rises	<14 rises
55–59 years old	<15 rises	<13 rises
60–64 years old	<14 rises	<12 rises
65–69 years old	<12 rises	<11 rises
70–74 years old	<12 rises	<10 rises
75–79 years old	<11 rises	<10 rises

**Table 2:** Sex grip strength dominant hand (kg) at admission.

			Grip Strength Dominant Hand (kg) at Admission			Total
			0–20 kg	20.1–35.5 kg	>35.5 kg	
Sex	Female	Count	12	38	1	51
		% of Total	14.3%	45.2%	1.2%	60.7%
	Male	Count	1	13	19	33
		% of Total	1.2%	15.5%	22.6%	39.3%
Total		Count	13	51	20	84
		% of Total	15.5%	60.7%	23.8%	100.0%
Chi-Square Tests						
		Value		df	*p*	
Pearson Chi-Square		35.537		2	0.000	

**Table 3:** Sex required time for five rises at admission (s).

			Required Time for Five Rises at Admission (s)				Total
			0–9 s	10–15 s	16–20 s	>20 s	
Sex	Female	Count	1	31	13	6	51
		% of Total	1.2%	36.9%	15.5%	7.1%	60.7%
	Male	Count	4	26	3	0	33
		% of Total	4.8%	31.0%	3.6%	0.0%	39.3%
Total		Count	5	57	16	6	84
		% of Total	6.0%	67.9%	19.0%	7.1%	100.0%
Chi-Square Tests							
			Value		df	*p*	
Pearson Chi-Square			11.143		3	0.011	

**Table 4:** Sex total number of rises in 30 s at admission.

			Total Number of Rises in 30 s at Admission				Total
			<5 Rises	5–10 Rises	11–15 Rises	>15 Rises	
Sex	Female	Count	1	26	23	1	51
		% of Total	1.2%	31.0%	27.4%	1.2%	60.7%
	Male	Count	0	6	26	1	33
		% of Total	0.0%	7.1%	31.0%	1.2%	39.3%
Total		Count	1	32	49	2	84
		% of Total	1.2%	38.1%	58.3%	2.4%	100.0%
Chi-Square Tests							
		Value		df		*p*	
Pearson Chi-Square		10.299		3		0.016	

**Table 5:** Age quadriceps muscle strength at discharge.

			Quadriceps Muscle Strength at Discharge				Total
			5	−5	4	−4	
Age	50–59	Count	32	8	0	0	40
		% of Total	38.1%	9.5%	0.0%	0.0%	47.6%
	60–69	Count	25	3	0	0	28
		% of Total	29.8%	3.6%	0.0%	0.0%	33.3%
	70–79	Count	10	3	2	1	16
		% of Total	11.9%	3.6%	2.4%	1.2%	19.0%
Total		Count	67	14	2	1	84
		% of Total	79.8%	16.7%	2.4%	1.2%	100.0%
Chi-Square Tests							
		Value		df		*p*	
Pearson Chi-Square		14.570		6		0.024	

**Table 6:** Age total number of rises in 30 s at discharge.

			Total Number of Rises in 30 s at Discharge				Total
			<5 Rises	5–10 Rises	11–15 Rises	>15 Rises	
Age	50–59	Count	1	9	28	2	40
		% of Total	1.2%	10.7%	33.3%	2.4%	47.6%
	60–69	Count	0	6	13	9	28
		% of Total	0.0%	7.1%	15.5%	10.7%	33.3%
	70–79	Count	0	10	5	1	16
		% of Total	0.0%	11.9%	6.0%	1.2%	19.0%
Total		Count	1	25	46	12	84
		% of Total	1.2%	29.8%	54.8%	14.3%	100.0%
Chi-Square Tests							
		Value		df		*p*	
Pearson Chi-Square		21.278		6		0.002	

**Table 7:** SARC-F total at admission quadriceps muscle strength at admission.

			Quadriceps Muscle Strength at Admission				Total
			5	−5	4	−4	
SARC-F Total at admission	0–3	Count	51	6	5	0	62
		% of Total	60.7%	7.1%	6.0%	0.0%	73.8%
	4–7	Count	8	5	3	1	17
		% of Total	9.5%	6.0%	3.6%	1.2%	20.2%
	8–12	Count	1	1	2	1	5
		% of Total	1.2%	1.2%	2.4%	1.2%	6.0%
Total		Count	60	12	10	2	84
		% of Total	71.4%	14.3%	11.9%	2.4%	100.0%
Chi-Square Tests							
	Value		df		*p*		
Pearson Chi-Square	21.466		6		0.002		

**Table 8:** SARC-F total at admission grip strength dominant hand (kg) at admission.

			Grip Strength Dominant Hand (kg) at Admission			Total
			0–20 kg	20.1–35.5 kg	>35.5 kg	
SARC-F Total at admission	0–3	Count	3	43	16	62
		% of Total	3.6%	51.2%	19.0%	73.8%
	4–7	Count	5	8	4	17
		% of Total	6.0%	9.5%	4.8%	20.2%
	8–12	Count	5	0	0	5
		% of Total	6.0%	0.0%	0.0%	6.0%
Total		Count	13	51	20	84
		% of Total	15.5%	60.7%	23.8%	100.0%
Chi-Square Tests						
		Value	df		*p*	
Pearson Chi-Square		35.363	4		0.000	

**Table 9:** SARC-F total at admission required time for five rises at admission (s).

			Required Time for Five Rises at Admission (s)				Total
			0–9 s	10–15 s	16–20 s	>20 s	
SARC-F Total at admission	0–3	Count	4	47	11	0	62
		% of Total	4.8%	56.0%	13.1%	0.0%	73.8%
	4–7	Count	1	10	3	3	17
		% of Total	1.2%	11.9%	3.6%	3.6%	20.2%
	8–12	Count	0	0	2	3	5
		% of Total	0.0%	0.0%	2.4%	3.6%	6.0%
Total		Count	5	57	16	6	84
		% of Total	6.0%	67.9%	19.0%	7.1%	100.0%
Chi-Square Tests							
		Value		df		*p*	
Pearson Chi-Square		32.336		6		0.000	

**Table 10:** SARC-F total at admission total number of rises in 30 s at admission.

			Total Number of Rises in 30 s at Admission				Total
			<5 Rises	5–10 Rises	11–15 Rises	>15 Rises	
SARC-F Total at admission	0–3	Count	0	19	42	1	62
		% of Total	0.0%	22.6%	50.0%	1.2%	73.8%
	4–7	Count	1	8	7	1	17
		% of Total	1.2%	9.5%	8.3%	1.2%	20.2%
	8–12	Count	0	5	0	0	5
		% of Total	0.0%	6.0%	0.0%	0.0%	6.0%
Total		Count	1	32	49	2	84
		% of Total	1.2%	38.1%	58.3%	2.4%	100.0%
Chi-Square Tests							
		Value		df		*p*	
Pearson Chi-Square		16.096		6		0.013	

**Table 11:** Spearman’s rank-order correlation between SARC-F (total) at admission and other parameters.

Nonparametric Correlations					
Correlations					
			Grip Strength Right Hand (kg) at Admission	Required Time for Five Rises at Admission (s)	Total Number of Rises in 30 s at Admission
Spearman’s rho	SARC-F Total at admission	Correlation Coefficient	−0.409	0.496	−0.484
		Sig. (2-tailed)	0.000	0.000	0.000
		N	84	84	84

**Table 12:** SARC-F total at discharge quadriceps muscle strength at discharge.

			Quadriceps Muscle Strength at Discharge				Total
			5	−5	4	−4	
SARC-F Total at discharge	0–3	Count	60	8	2	0	70
		% of Total	71.4%	9.5%	2.4%	0.0%	83.3%
	4–7	Count	4	6	0	1	11
		% of Total	4.8%	7.1%	0.0%	1.2%	13.1%
	8–12	Count	3	0	0	0	3
		% of Total	3.6%	0.0%	0.0%	0.0%	3.6%
Total		Count	67	14	2	1	84
		% of Total	79.8%	16.7%	2.4%	1.2%	100.0%
Chi-Square Tests							
		Value	df		*p*		
Pearson Chi-Square		21.221	6		0.002		

**Table 13:** SARC-F total at discharge grip strength dominant hand (kg) at discharge.

			Grip Strength Dominant Hand (kg) at Discharge			Total
			0–20 kg	20.1–35.5 kg	>35.5 kg	
SARC-F Total at discharge	0–3	Count	8	40	22	70
		% of Total	9.5%	47.6%	26.2%	83.3%
	4–7	Count	4	6	1	11
		% of Total	4.8%	7.1%	1.2%	13.1%
	8–12	Count	3	0	0	3
		% of Total	3.6%	0.0%	0.0%	3.6%
Total		Count	15	46	23	84
		% of Total	17.9%	54.8%	27.4%	100.0%
Chi-Square Tests						
		Value	df		*p*	
Pearson Chi-Square		19.365	4		0.001	

**Table 14:** SARC-F total at discharge required time for five rises at discharge (s).

			Required Time for Five Rises at Discharge (s)				Total
			0–9 s	10–15 s	16–20 s	>20 s	
SARC-F Total at discharge	0–3	Count	18	47	4	1	70
		% of Total	21.4%	56.0%	4.8%	1.2%	83.3%
	4–7	Count	0	6	3	2	11
		% of Total	0.0%	7.1%	3.6%	2.4%	13.1%
	8–12	Count	0	1	0	2	3
		% of Total	0.0%	1.2%	0.0%	2.4%	3.6%
Total		Count	18	54	7	5	84
		% of Total	21.4%	64.3%	8.3%	6.0%	100.0%
Chi-Square Tests							
		Value		df		*p*	
Pearson Chi-Square		33.608		6		0.000	

**Table 15:** SARC-F total at discharge total number of rises in 30 s at discharge.

			Total Number of Rises in 30 s at Discharge				Total
			<5 Rises	5–10 Rises	11–15 Rises	>15 Rises	
SARC-F Total at discharge	0–3	Count	0	15	43	12	70
		% of Total	0.0%	17.9%	51.2%	14.3%	83.3%
	4–7	Count	1	7	3	0	11
		% of Total	1.2%	8.3%	3.6%	0.0%	13.1%
	8–12	Count	0	3	0	0	3
		% of Total	0.0%	3.6%	0.0%	0.0%	3.6%
Total		Count	1	25	46	12	84
		% of Total	1.2%	29.8%	54.8%	14.3%	100.0%
Chi-Square Tests							
		Value		df		*p*	
Pearson Chi-Square		23.612		6		0.001	

**Table 16:** Spearman’s rank-order correlation between SARC-F (total) at discharge and other parameters.

			Grip Strength Dominant Hand (kg) at Discharge	Required Time for Five Rises at Discharge (s)	Total Number of Rises in 30 s at Discharge
Spearman’s rho	SARC-F Total at discharge	Correlation Coefficient	−0.494	0.378	−0.479
		Sig. (2-tailed)	0.000	0.000	0.000
		N	84	84	84

## Data Availability

The data presented in this study are available on valid request from the corresponding authors. The data are not publicly available due to privacy and ethical concerns.
